# Germline variants of homology‐directed repair or mismatch repair genes in cervical cancer

**DOI:** 10.1002/ijc.35221

**Published:** 2024-10-23

**Authors:** Lara Kokemüller, Dhanya Ramachandran, Peter Schürmann, Robert Geffers, Matthias Jentschke, Gerd Böhmer, Hans‐Georg Strauß, Christine Hirchenhain, Monika Schmidmayr, Florian Müller, Peter A. Fasching, Alexander Luyten, Norman Häfner, Peter Hillemanns, Thilo Dörk

**Affiliations:** ^1^ Department of Gynaecology Hannover Medical School Hannover Germany; ^2^ Genome Analytics Helmholtz Centre for Infection Research Braunschweig Germany; ^3^ IZD Ärztliche Partnerschaft Böhmer & Partner Hannover Germany; ^4^ Gynaecology Department Martin‐Luther‐University Halle‐Wittenberg Halle Saale Germany; ^5^ Gynaecology Department University Hospital Carl Gustav Carus Dresden Dresden Germany; ^6^ Gynaecology Department Technical University of Munich Munich Germany; ^7^ Gynaecology Department Martin Luther Hospital Berlin Germany; ^8^ Department of Gynaecology and Obstetrics, University Hospital Erlangen Friedrich Alexander University of Erlangen‐Nuremberg Erlangen Germany; ^9^ Department of Gynaecology and Obstetrics MARE Klinikum, Dysplasia Unit Kronshagen Germany; ^10^ Clinics of Gynaecology and Obstetrics Wolfsburg Hospital Wolfsburg Germany; ^11^ Department of Gynaecology Friedrich Schiller University Jena Jena Germany

**Keywords:** cervical carcinoma, homologous recombination, human papillomavirus, mismatch repair, targeted sequencing

## Abstract

While cervical cancer is associated with a persistent human papillomavirus (HPV) infection, the progression to cancer is influenced by genomic risk factors that have remained largely obscure. Pathogenic variants in genes of the homology‐directed repair (HDR) or mismatch repair (MMR) are known to predispose to diverse tumour entities including breast and ovarian cancer (HDR) or colon and endometrial cancer (MMR). We here investigate the spectrum of HDR and MMR germline variants in cervical cancer, with particular focus on the HPV status and histological subgroups. We performed targeted next‐generation sequencing for 5 MMR genes and 12 HDR genes on 728 German patients with cervical dysplasia or invasive cancer. In total, 4% of our patients carried a pathogenic germline variant, based on ClinVar classifications and additional ESM1b and AlphaMissense predictions. These included 15 patients with truncating variants in HDR genes (*BARD1*, *BRCA1*, *BRCA2*, *BRIP1*, *FANCM*, *RAD51D* and *SLX4*). MMR‐related gene variants were less prevalent and mainly of the missense type. While MMR‐related gene variants tended to associate with adenocarcinomas, HDR gene variants were commonly observed in squamous cancers. While one patient with HPV‐negative cancer carried a pathogenic MMR gene variant (in *MSH6*), the HDR germline variants were found in patients with HPV‐positive cancers and tended to associate with HPV18. Taken together, our study supports a potentially risk‐modifying role of MMR and HDR germline variants in cervical cancer but no association with HPV‐negative status. These variants may be exploitable in future therapeutic managements.

## Graphical abstract

What's new?

Pathogenic variants in genes associated with DNA repair may influence the progression of cervical cancer. Here, the authors analysed 12 homology‐directed repair (HDR) and 5 mismatch repair (MMR) genes in 728 patients with cervical dysplasia or invasive cancer. They performed targeted sequencing and found that 4% of patients carried germline variants with known or predicted pathogenicity in these genes. The variants did not contribute significantly to HPV‐negative cervical cancer, but HDR variants were associated with HPV18‐positive cervical cancer and MMR variants were associated with adenocarcinomas.

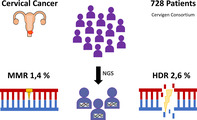

## 1 INTRODUCTION

Cervical cancer represents the fourth most frequent cancer among women worldwide and accounts for one of the most common cancer‐related deaths in women.^1^ Persistent viral infection of high‐risk human papillomavirus (HPV) types is the most important contributor to cervical cancer development.^2^ HPV infection leads to uncontrolled cell proliferation and progress in carcinogenesis due to interference with tumour suppressor proteins and immune response.^2^, ^3^ However, some 90% of the cases resolve the infection within 2 years and only a subset progresses towards invasive disease.^4^ The decisive factors in this process are incompletely understood. Furthermore, some 3%–8% of cases appear to develop without detectable HPV infection and show distinct pathological and molecular features and a worse prognosis.^5^, ^6^ It is unknown whether these cancers may have arisen from a genetic predisposition. Although large multiple‐case families are rare in cervical cancer, an about twofold increased familial relative risk has been estimated from national cancer registries.^7^

Hereditary cancer syndromes are often caused by pathogenic variants affecting DNA repair pathways, with homology‐directed repair (HDR) or mismatch repair (MMR) being the most relevant for gynaecological cancers. HDR recovers DNA double strand breaks (DSB) by taking sister chromatids as template.^8^ Subsequent repair occurs through homologous recombination or single‐strand annealing. Compared with other DSB repair mechanisms, HDR works largely error‐free in maintaining genomic stability.^8^ Pathogenic germline variants in HDR genes have been linked to hereditary breast and ovarian cancer (HBOC). On the other hand, mismatch repair is safeguarded by the products of the genes *MLH1*, *MSH2*, *MSH6* and *PMS2*, which synergise to correct mismatched bases, insertions and deletions within short repeats, also called microsatellites.^9^ Inactivation of this repair pathway results in microsatellite instability (MSI) and hypermutability that can further oncogenic processes.^9^ Pathogenic germline variants in MMR genes cause Lynch Syndrome, one of the most prevalent hereditary cancer syndromes with a particularly high risk for endometrial and colorectal cancers.^10^

While the role of HDR or MMR germline variants in breast, ovarian or endometrial cancers have been well elaborated, their impact on cervical cancer is less clear. In the present study, we investigate a large cervical cancer series for the prevalence of pathogenic variants in 5 MMR and 12 HDR genes and their potential association with HPV status and tumour characteristics.

## 2 METHODS

### 2.1 Patient cohort for sequencing

Genomic DNA samples for the present study were selected from the case–control series of patients with cervical cancer or dysplasia of the German Cervigen Consortium.^11^ We intentionally enriched the patient cohort for invasive cancer and included all available patients with HPV‐undetected cervical cancer into the sequencing study. An overview of the age, histology and HPV distribution is provided in Table S1.

### 2.2 Sequencing analysis

Peripheral blood samples from the initially 734 patients were used for DNA extraction with standard phenol chloroform extraction. Target‐specific primers were designed through the Fluidigm D3 online platform (www.d3.fluidigm.com) (Fluidigm, USA) for four selected MMR genes (*MLH1*, *MSH2*, *MSH6*, *PMS2*), one MMR‐related gene (*MUTYH*) and 12 HDR genes (*BARD1*, *BRCA1*, *BRCA2*, *BRIP1*, *ERCC4*, *FANCM*, *PALB2*, *RAD51*, *RAD51B*, *RAD51C*, *RAD51D* and *SLX4*). The primers were designed to generate amplicons of 300–500 bp and provided about 90% coverage of the target regions (Table S2). Multiplex polymerase chain reaction (PCR) was performed with 250 ng DNA, primers and sample‐specific barcodes using the LP 48.48 Integrated Fluidic Circuit Access array (Fluidigm, USA) on a Fluidigm FC1 Cycler following the manufacturer's protocol. PCR products were pooled and purified utilising AMPure XP magnetic beads. Specific adaptors were added for sequencing the pooled library on an Illumina MiSeq 500 system (Illumina, USA). The MiSeq Reagent Kit (Illumina, USA) was used for paired‐end sequencing. Sequencing quality was checked by the Q30 scores and samples with scores >85% were considered successful. We eliminated six samples due to insufficient coverage and remained with 728 sequenced samples for the downstream analyses. The total number of reads and coverage per sample for these 728 patients are provided in Table S3.

### 2.3 Variant annotation and classification

Sequencing results as fastq files were aligned to gene specific .gbk files (human GrCh37), annotated and analysed using NextGENe 4.2.2 (SoftGenetics, USA). Alignment parameters were matching requirements ≥80% within greater than 30 bases; mutation percentage ≤25%; SNP allele counts ≤5 and total coverage counts ≤15.

Initial classification of variants was achieved with Mutation taster (www.mutationtaster.org) using Variant Call Format (VCF) files for each sample. Polymorphic variants, sequencing artefacts and pseudogene variants for *PMS2* were removed through filtering for minor allele frequency (MAF) < 0.005. We then used the ClinVar database (https://www.ncbi.nlm.nih.gov/clinvar/; accessed on 16 May 2024) to classify known variants by pathogenicity. For novel variants, we classified truncating variants as pathogenic if they did not affect the last exon. The same criterion was applied to a control data set derived from the UK Biobank as accessed via the GeneBass browser (https://app.genebass.org/) and to a second control data set derived from the Regeneron Genetics Center (RGC) as accessed via the RGC Million Exome (RGC‐ME) Variant browser (https://rgc-research.regeneron.com/me/). Individuals in the UK Biobank were largely of European descent (94%) similar to our study and encompassed 4529 phenotypes, including a history of any cancer in some 11% of individuals.^12^ Individuals in the RGC‐ME database were more ethnically diverse but still included 76.7% Europeans.^13^ Canonical splice‐site variants were classified based on MaxEntScan prediction (http://hollywood.mit.edu/burgelab/maxent/Xmaxentscan_scoreseq.html). Missense variants of uncertain significance or without ClinVar classification were additionally analysed using the Evolutionary Scale Modeling 1b (ESM1b; https://huggingface.co/spaces/ntranoslab/esm_variants) and AlphaMissense algorithms (https://github.com/deepmind/alphamissense). For ESM1b prediction, a threshold score − 10 was set for pathogenicity. For AlphaMissense, we used a score >0.8 as evidence of pathogenicity. Missense variants were classified as likely pathogenic or likely benign if both ESM1b and AlphaMissense concurred in their prediction. Where ESM1b and AlphaMissense predictions were discordant, the variant remained ambiguous and was not considered as a pathogenic variant.

### 2.4 Sanger sequencing for validation

Pathogenic variants were validated using Sanger sequencing in the corresponding patient DNA sample. Exon‐specific primers were used (Table S4) to generate the respective PCR products using HotStar T*aq* DNA polymerase (Qiagen, Germany). Products were sequenced using the Big Dye Terminator Kit v1.1 (Applied Biosystems) and separated through capillary gel electrophoresis on a Seq Studio Genetic Analyzer (Applied Biosystems). Electropherograms were analysed using Finch TV (Geospiza Inc.) (Figure S1).

### 2.5 Statistical analysis

We used Fisher's exact test to compare carrier frequencies between subgroups. Distribution of age at diagnosis was compared between groups using a median test in STATA17 that is based on Pearson's correlation with continuity correction. *p* values were two‐sided, and *p* < .05 was considered to indicate evidence of association.

## 3 RESULTS

The sequencing of genomic DNA samples from 728 patients with cervical dysplasia or invasive carcinoma in the coding regions of 17 candidate genes revealed 23 pathogenic/likely pathogenic germline variants of the MMR genes *MLH1*, *MSH2* and *MSH6*, the MMR‐related gene *MUTYH* or the HDR genes *BARD1*, *BRCA1*, *BRCA2*, *BRIP1*, *FANCM*, *RAD51D* and *SLX4* in a total of 29 patients (4.0%) (Table 1). Most carriers had pathogenic variants in HDR genes (17/23, 74%), and most of the variants were truncating. Thirteen variants had been classified as pathogenic in ClinVar. A total of 39 variants had been listed with uncertain significance or were novel and were thus subjected to further classification through ESM1b and AlphaMissense predictions. This led to a likely pathogenic classification for further four variants while predictions for seven variants remained ambiguous under strict thresholds, and 27 variants were classified as likely benign (Table 1). The remaining splice‐site variant in *RAD51B*, rs148518198, displayed only a slight decrease in the MaxENT score from 10.57 to 8.38, which was also not considered pathogenic. Four pathogenic germline variants in HDR genes (two in *SLX4*, one in *BRCA2* and one in *FANCM*) had not been reported previously (Table 1; Figure S1).

**TABLE 1:** Rare germline variants of MMR and HDR genes in cervical cancer.

Gene	Chrom	Pos	Nucleotide variation	Change in mRNA	Change in protein	Variation ID	No. of patients	Molecular consequence	ClinVar prediction
HDR genes									
Truncating/splicing									
*BARD1*	2	215,610,566	NC_000002.11:g.215610566G > A	NM_000465.4:c.1690C > T	NP_000456.2:p.Gln564Ter	rs587780021	1	Stop gained	Pathogenic
*BRCA1*	17	41,245,598	NC_000017.10:g.41245595_41245598delCTTT	NM_007294.4:c.1953_1956delCTTT	NP_009225.1:p.Lys653fs	rs80357526	1	Frameshift	Pathogenic
*BRCA1*	17	41,245,583	NC_000017.10:g.41245583G > T	NM_007294.4:c.1965C > A	NP_009225.1:p.Tyr655Ter	rs886039987	1	Stop gained	Pathogenic
*BRCA2*	13	32,914,769	NC_000013.10:g.32914769delCA	NM_000059.4:c.6277delCA	NP_000050.3:p.His2093fs	novel	1	Frameshift	Not listed
*BRCA2*	13	32,912,966	NC_000013.10:g.32912966delAAAG [1]	NM_000059.4:c.4478_4481del	NP_000050.3:p.Glu1493fs	rs80359454	1	Frameshift	Pathogenic
*BRIP1*	17	59,926,513	NC_000017.10:g.59926513G > A	NM_032043.3:c.484C > T	NP_114432.2:p.Arg162Ter	rs747604569	1	Stop gained	Pathogenic/likely pathogenic
*BRIP1*	17	59,820,480	NC_000017.10:g.59820480dupA	NM_032043.3:c.2273dup	NP_114432.2:p.Ala759fs	rs587780236	1	Frameshift	Pathogenic/likely pathogenic
*BRIP1*	17	59,761,416	NC_000017.10:g.59761414TTTG [2]	NM_032043.3:c.2990_2993delCAAA	NP_114432.2:p.Thr997fs	rs771028677	1	Frameshift	Pathogenic/likely pathogenic
*FANCM*	14	45,658,326	NC_000014.8:g.45658326C > T	NM_020937.4:c.5101C > T	NP_065988.1:p.Gln1701Ter	rs147021911	3	Stop gained	Pathogenic/likely pathogenic
*FANCM*	14	45,636,336	NC_000014.8:g.45636336C > T	NM_020937.4:c.1972C > T	NP_065988.1:p.Arg658Ter	rs368728266	1	Stop gained	Pathogenic/likely pathogenic
*RAD51D*	17	33,428,366	NC_000017.10:g.33428366G > A	NM_002878.4:c.757C > T	NP_002869.3:p.Arg253Ter	rs137886232	1	Stop gained	Pathogenic
*SLX4*	16	3,641,255	NC_000016.9:g.3641255G > C	NM_032444.4:c.2384C > G	NP_115820.2:p.Ser795Ter	rs2151126006	1	Stop gained	Pathogenic/likely pathogenic
*SLX4*	16	3,639,616	NC_000016.9:g.3639616delT	NM_032444.4:c.4024delA	NP_115820.2:p.Ser1342fs	novel	1	Frameshift	Not listed
Alpha missense /ESM1b pathogenic									
*BRIP1*	17	59,820,468	NC_000017.10:g.59820468C > T	NM_032043.3:c.2285G > A	NP_114432.2:p.Arg762His	rs200960251	1	Missense	Uncertain significance
*FANCM*	14	45,605,574	NC_000014.8:g.45605574G > A	NM_020937.4:c.340G > A	NP_065988.1:p.Gly114Arg	rs376195946	1	Missense	Not listed
*FANCM*	14	45,606,344	NC_000014.8:g.45606344G > A	NM_020937.4:c.581G > A	NP_065988.1:p.Met194Thr	novel	1	Missense	Not listed
*SLX4*	16	3,641,087	NC_000016.9:g.3641087A > C	NM_032444.4:c.2552 T > G	NP_115820.2:p.Met851Arg	novel	1	Missense	Not listed
Alpha missense /ESM1b ambiguous									
*BRIP1*	17	59,876,546	NC_000017.10:g.59876546G > A	NM_032043.3:c.1255C > T	NP_114432.2:p.Arg419Trp	rs150624408	2	Missense	Uncertain significance
*BRIP1*	17	59,937,223	NC_000017.10:g.59937223G > C	NM_032043.3:c.139C > G	NP_114432.2:p.Pro47Ala	rs28903098	1	Missense	Uncertain significance
*ERCC4*	16	14,041,848	NC_000016.9:g.14041848C > T	NM_005236.3:c.2395C > T	NP_005227.1:p.Arg799Trp	rs121913049	1	Missense	Uncertain significance/likely pathogenic
*FANCM*	14	45,623,908	NC_000014.8:g.45623908C > T	NM_020937.4:c.1192C > T	NP_001295062.1:p.Arg372Trp	rs752364451	1	Missense	Uncertain significance
*PALB2*	16	23,646,617	NC_000016.9:g.23646617G > T	NM_024675.4:c.1250C > A	NP_078951.2:p.Ser417Tyr	rs45510998	1	Missense	Uncertain significance/likely benign
*RAD51*	15	41,021,831	NC_000015.9:g.41021831A > C	NM_002875.5:c.773A > C	NP_002866.2:p.Glu258Ala	rs191297852	1	Missense	Not listed
*RAD51C*	17	56,774,077	NC_000017.10:g.56774077A > G	NM_058216.3:c.428A > G	NP_478123.1:p.Gln143Arg	rs587780255	1	Missense	Uncertain significance
Alpha missense /ESM1b benign									
*BARD1*	2	215,632,251	NC_000002.11:g.215632251 T > C	NM_000465.4:c.1523A > G	NP_000456.2:p.Asp508Gly	rs769529578	1	Missense	Uncertain significance
*BRCA1*	17	41,215,920	NC_000017.10:g.41215920G > A	NM_007294.4:c.5123C > T	NP_009225.1:p.Ala1708Val	rs28897696	1	Missense	Uncertain significance
*BRCA1*	17	41,245,297	NC_000017.10:g.41245297 T > A	NM_007294.4:c.2251A > T	NP_009225.1:p.Met751Leu	novel	1	Missense	Not listed
*BRCA1*	17	41,245,762	NC_000017.10:g.41245762G > C	NM_007300.4:c.1786C > G	NP_009225.1:p.Leu596Val	rs80357371	1	Missense	Uncertain significance
*BRCA2*	13	32,893,453	NC_000013.10:g.32893457_32893462delACTTAG	NM_000059.4:c.311_316del	NP_000050.3:p.Asp104_Leu105del	rs2138705876	1	In frame deletion	Uncertain significance
*BRCA2*	13	32,907,440	NC_000013.10:g.32907440C > G	NM_000059.4:c.1825C > G	NP_000050.3:p.Gln609Glu	rs80358472	1	Missense	Uncertain significance
*BRCA2*	13	32,912,456	NC_000013.10:g.32912458_32912460delCAA	NM_000059.4:c.3966_3968delCAA	NP_000050.3:p.Asn1322del	rs397507319	1	In frame deletion	Uncertain significance
*BRCA2*	13	32,914,540	NC_000013.10:g.32914543_32914548delAAGTAA	NM_000059.4:c.6051_6056delAAGTAA	NP_000050.3:p.Lys2017_Ser2018del	rs2072545044	1	In frame deletion	Uncertain significance
*BRCA2*	13	32,918,695	NC_000013.10:g.32918695G > A	NM_000059.4:c.6842G > A	NP_000050.3:p.Gly2281Glu	rs80358908	1	Missense	Uncertain significance
*BRCA2*	13	32,972,745	NC_000013.10:g.32972745delinsGAATTATATCT	NM_000059.4:c.10095delinsGAATTATATCT	NP_000050.3:p.Ser3366fs	rs276174803	1	Frameshift (last exon)	Uncertain significance/benign/likely benign
*ERCC4*	16	14,015,936	NC_000016.9:g.14015936C > T	NM_005236.3:c.256C > T	NP_005227.1:p.Arg86Cys	rs769932063	1	Missense	Uncertain significance
*ERCC4*	16	14,028,147	NC_000016.9:g.14028147C > T	NM_005236.3:c.1201C > T	NP_005227.1:p.Leu401Phe	rs147458778	1	Missense	Uncertain significance
*ERCC4*	16	14,029,366	NC_000016.9:g.14029366C > T	NM_005236.3:c.1577C > T	NP_005227.1:p.Pro526Leu	rs149056863	1	Missense	Uncertain significance
*ERCC4*	16	14,041,882	NC_000016.9:g.14041882C > T	NM_005236.3:c.2429C > T	NP_005227.1:p.Ala810Val	rs748683649	1	Missense	Not listed
*ERCC4*	16	14,041,905	NC_000016.9:g.14041905C > G	NM_005236.3:c.2452C > G	NP_005227.1:p.Gln818Glu	rs774635437	1	Missense	Uncertain significance
*FANCM*	14	45,605,493	NC_000014.8:g.45605493A > G	NM_020937.4:c.259A > G	NP_065988.1:p.Thr87Ala	novel	1	Missense	Not listed
*FANCM*	14	45,645,364	NC_000014.8:g.45645364 T > C	NM_020937.4:c.3407 T > C	NP_065988.1:p.Leu1136Ser	rs770989272	1	Missense	Uncertain significance
*PALB2*	16	23,641,593	NC_000016.9:g.23641585_23641593delGCAGGACTT	NM_024675.4:c.1882_1890del	NP_078951.2:p.Lys628_Cys630del	rs587778583	1	In frame deletion	Uncertain significance
*PALB2*	16	23,646,722	NC_000016.9:g.23646722C > T	NM_024675.4:c.1145G > A	NP_078951.2:p.Ser382Asn	rs515726063	1	Missense	Uncertain significance
*RAD51B*	14	68,331,763	NC_000014.8:g.68331763 T > C	NM_002877.6:c.359 T > C	NP_002868.1:p.Met120Thr	rs142567687	1	Missense	Not listed
*RAD51B*	14	68,331,840	NC_000014.8:g.68331840G > A	NM_002877.6:c.436G > A	NP_002868.1:p.Ala146Thr	rs200741476	1	Missense	Uncertain significance
*RAD51D*	17	33,428,351	NC_000017.10:g.33428351C > T	NM_133629.3:c.436G > A	NP_598332.1:p.Gly146Arg	rs181695922	1	Missense	Uncertain significance
*SLX4*	16	3,640,291	NC_000016.9:g.3640291C > G	NM_032444.4:c.3348G > C	NP_115820.2:p.Met1116Ile	novel	1	Missense	Not listed
*SLX4*	16	3,640,328	NC_000016.9:g.3640328C > T	NM_032444.4:c.3311G > A	NP_115820.2:p.Arg1104Gln	rs767093188	1	Missense	Not listed
*SLX4*	16	3,640,620	NC_000016.9:g.3640620G > T	NM_032444.4:c.3019C > A	NP_115820.2:p.Gln1007Lys	rs138798067	1	Missense	Uncertain significance
*SLX4*	16	3,642,722	NC_000016.9:g.3642722C > G	NM_032444.4:c.2305G > C	NP_115820.2:p.Glu769Gln	rs150712805	1	Missense	Uncertain significance
*SLX4*	16	3,656,486	NC_000016.9:g.3656486A > G	NM_032444.4:c.749 T > C	NP_115820.2:p.Met250Thr	rs950041115	1	Missense	Not listed
MaxEntScore benign									
*RAD51B*	14	68,934,972	NC_000014.8:g.68934972G > A	NM_002877.6:c.1036 + 5G > A	‐	rs148518198	1	Donor splice‐site variant	Uncertain significance
MMR genes									
Truncating									
*MUTYH*	1	45,797,374	NC_000001.10:g.45797374delG	NM_001048174.2:c.1063del	NP_001041639.1:p.Ala357fs	rs587778536	1	Frameshift	Pathogenic
Pathogenic missense									
*MUTYH*	1	45,797,228	NC_000001.10:g.45797228C > T	NM_001048174.2:c.1103G > A	NP_001041639.1:p.Gly368Asp	rs36053993	5	Missense	Pathogenic/likely pathogenic
*MUTYH*	1	45,798,475	NC_000001.10:g.45798475 T > C	NM_001048174.2:c.452A > G	NP_001041639.1:p.Tyr151Cys	rs34612342	1	Missense	Pathogenic
Alpha missense /ESM1b pathogenic									
*MLH1*	3	37,083,793	NC_000003.11:g.37083793 T > G	NM_000249.4:c.1702 T > G	NP_000240.1:p.Phe568Val	novel	1	Missense	Not listed
*MSH2*	2	47,637,405	NC_000002.11:g.47637405A > G	NM_000251.3:c.539A > G	NP_000242.1:p.Asp180Gly	novel	1	Missense	Not listed
*MSH6*	2	48,032,823	NC_000002.11:g.48032823C > A	NM_000179.3:c.3623C > A	NP_000170.1:p.Ser1208Tyr	rs1572742021	1	Missense	Uncertain significance
alpha missense /ESM1b ambiguous									
*MUTYH*	1	45,797,444	NC_000001.10:g.45797444G > T	NM_001048174.2:c.991C > A	NP_001041639.1:p.Pro331Thr	rs587782773	1	Missense	Uncertain significance
*PMS2*	7	6,017,284	NC_000007.13:g.6017284G > A	NM_000535.7:c.2380C > T	NP_000526.2:p.Pro794Ser	rs773393960	1	Missense	Uncertain significance
*PMS2*	7	6,038,824	NC_000007.13:g.6038824C > T	NM_000535.7:c.620G > A	NP_000526.2:p.Gly207Glu	rs374704824	1	Missense	Uncertain significance/likely benign
Alpha missense /ESM1b benign									
*MLH1*	3	37,056,023	NC_000003.11:g.37056023C > T	NM_000249.4:c.778C > T	NP_000240.1:p.Leu260Phe	rs63750642	1	Missense	Uncertain significance
*MLH1*	3	37,089,173	NC_000003.11:g.37089173A > G	NM_000249.4:c.1895A > G	NP_000240.1:p.Glu632Gly	rs1064793638	1	Missense	Uncertain significance
*MUTYH*	1	45,797,846	NC_000001.10:g.45797846G > A	NM_001048174.2:c.841C > T	NP_001041639.1:p.Arg281Cys	rs138089183	1	Missense	Uncertain significance
*PMS2*	7	6,037,049	NC_000007.13:g.6037049 T > G	NM_000535.7:c.711A > C	NP_000526.2:p.Gln237His	rs368608818	1	Missense	Not listed

*Note*: Rare germline variants of mismatch repair (MMR) and homology‐directed repair (HDR) genes identified through targeted amplicon sequencing of 17 genes in 728 patients with cervical cancer. Variants are listed by their chromosomal position according to the GRCh37.p13 genome build and are annotated using the National Center for Biotechnology Information (NCBI) reference gene, NM transcript and NP protein isoform as indicated. Variation ID refers to the NCBI SNP database (https://www.ncbi.nlm.nih.gov/snp/), and ClinVar prediction refers to the NCBI ClinVar database (https://www.ncbi.nlm.nih.gov/clinvar/; accessed on 16 May 2024). Missense variants in HDR or MMR genes were categorised as pathogenic when both ESM1b and AlphaMissense predictions were pathogenic or were categorised as benign when both ESM1b and AlphaMissense predictions were benign. Discordant predictions are listed as ‘alpha missense/ESM1b ambiguous’. Donor splice‐site variant c.1036 + 5G > A in *RAD51B* was assessed through MaxEntScan prediction (http://hollywood.mit.edu/burgelab/maxent/Xmaxentscan_scoreseq.html). Chrom, chromosome; Pos, position in genome build 37; accession prefixes NC_, NM_ and NP_ refer to the curated eukaryotic reference sequences for chromosome, mRNA and protein, respectively.

We noticed that 15 patients carried truncating variants in HDR genes, including four in *FANCM*, three in *BRIP1*, two each in *BRCA1*, *BRCA2*, *SLX4,* and one each in *BARD1* and *RAD51D*. Truncating variants in *BRCA1*, *BRCA2*, *BRIP1* and *RAD51D* represent high‐risk variants for breast or ovarian cancer. Overall, the cumulative frequency of germline truncating HDR gene variants in cervical cancer patients appeared slightly higher than expected from their prevalence among population‐based controls derived from either the UK Biobank (odds ratio [OR], 1.25; 95% confidence interval [CI], 0.70–2.07; *p* = .38) or the RGC‐ME dataset (OR, 1.39; 95% CI, 0.78–2.30; *p* = .22) but this difference was not statistically significant and the number of carriers in our study was too small for a gene‐by‐gene analysis (Table S5).^12^, ^13^

We investigated whether MMR or HDR germline variants were associated with distinct histological subtypes, earlier age at diagnosis or family history of cancer. Pathogenic HDR variants were mostly found in squamous cell carcinoma but not significantly more often than for non‐carriers (OR, 1.53; *p* = .59). MMR germline variants occurred about fourfold more often in adenocarcinoma (*p* = .07 or *p* = .01 without or with ambiguous variants included, respectively). Specifically, the *MSH2* and *MSH6* pathogenic variant carriers and four of the five carriers of *MUTYH* missense variant p.G368D had adenocarcinoma. Patients with pathogenic variants tended to have an earlier age at diagnosis of invasive cervical cancer compared with non‐carriers (mean age, 42.2 years; 95% CI, 38.1–46.3 years, in MMR carriers and 46.0 years; 95% CI, 38.1–53.9 years, in HDR variant carriers, compared to 47.3 years, 95% CI, 46.1–48.5 years, in non‐carriers with invasive cancer), but this difference was not statistically significant (*p* = .2 and *p* = .9 for MMR and HDR, respectively). Family history of cancer was documented only for six pathogenic variant carriers (four HDR, two MMR). One *BRCA2* variant carrier reported a second‐degree family history for cervical cancer and potential ovarian cancer, and one *FANCM* variant carrier reported a second‐degree family history of breast cancer.

We also investigated whether MMR or HDR gene variants were enriched in patients with particular HPV types. We found two patients with HPV‐negative invasive cancer and pathogenic missense variants in the MMR genes *MSH6* and *MUTYH*, respectively, and another patient with HPV‐negative dysplasia and a pathogenic variant in *FANCM*. However, the large majority of pathogenic variants in both MMR and HDR genes were found in HPV‐positive patients. When invasive cancers were stratified by HPV type, 6 of 12 carriers of pathogenic HDR variants had an HPV18‐positive status, which was about threefold more than in non‐carriers (OR, 2.97; *p* = .09, 2 df, Fisher's exact test). The potential association with HPV18‐positive invasive cancers became slightly stronger when only truncating HDR gene variants were considered (OR, 4.95; *p* = .03, 2 df, Fisher's exact test).

## 4 DISCUSSION

Genital infection with HPV is seminal but not sufficient in the aetiology of cervical cancer, and hereditary factors are thought to play an important role. We investigated a potential impact of genomic MMR and HDR gene variants on cervical cancer risk, also considering HPV status and histology. MMR and HDR gene variants are known predisposing factors for endometrial cancers and for breast and ovarian cancers, respectively, but have not extensively been investigated in cervical cancer. However, a recent study reported about 6.4% pathogenic or likely pathogenic germline variants in 358 Chinese cervical cancer patients, mostly in HDR genes.^14^ Interestingly, that study proposed a significant association of pathogenic variants in *BRCA1* or *BRCA2* with cervical cancer (*BRCA1*: OR, 4.92; *p* = .01; *BRCA2*: OR, 4.46; *p* = .02) compared with an East Asian reference population in gnomAD.^14^ The estimate for *BRCA1* would be consistent with an early study of the Breast Cancer Linkage Consortium that reported a significantly increased risk for cervical cancer in *BRCA1* mutation carriers (RR, 3.72; *p* < .001).^15^ It has remained unknown whether these cancers associated with a specific HPV status or histology.

Our study identified 19/728 carriers (2.6%) of pathogenic HDR gene variants, including 15 truncating variants, of which 4 were in *BRCA1* or *BRCA2* (0.5%) and 4 were in *BRIP1* or *RAD51D*, two other genes associated with high ovarian cancer risk. Overall, the frequency of truncating HDR gene variants in our cervical cancer series appeared slightly higher than expected from population‐based controls as represented in the large UK Biobank or Regeneron resources.^12^, ^13^ While this did not reach statistical significance, our findings confirm that a substantial proportion of cervical cancer patients harbour pathogenic HDR gene variants, in line with the results by Wen et al.^14^ Interestingly, this fraction of cases did not explain HPV‐negative cervical cancer because all HDR variants except one (a *FANCM* variant) were identified in HPV‐positive cases in our study. We found modest evidence of an association with HPV18‐positive invasive carcinoma, in particular for truncating HDR gene variants. In regard of HDR variants in HPV‐positive carcinomas, it is interesting to note that Fanconi anaemia patients have a high susceptibility to HPV‐induced oral or anogenital carcinomas including cervical cancer, and several HDR genes including *BRCA1*, *BRCA2*, *BRIP1* and *SLX4* are also Fanconi anaemia genes.^16^ Furthermore, it has been observed by Wen et al. that HDR variant carriers among cervical cancer patients were younger than non‐carriers (age at diagnosis 46 compared with 52 years; *p* = .04). While the latter could not be statistically confirmed in our study, the potential associations with HPV subtype, histology and potentially age suggest that HDR gene variants may not be merely innocent bystanders but could take an active role in driving tumorigenesis towards invasive cervical carcinoma.

An association of cervical cancer and Lynch syndrome has also not been firmly established although MMR gene variant carriers were reported with an about sixfold higher risk and a 4 years earlier diagnosis of cervical cancer compared with the general population.^17^ The corresponding cumulative risk to 80 years was 4.5% compared with 0.8% for the general population.^17^ HPV‐negative endocervical carcinomas in Lynch syndrome have been reported in sisters with a *MSH2* gene variant.^18^ It has been suggested that some HPV‐negative cases may represent lower uterine segment endometrial cancers involving the endocervix or may include cancers with uncertain pathological assignment while on the other hand it has also been suggested that Lynch Syndrome may be associated with non‐HPV related cervical adenocarcinomas and some endocervical cancers may be misclassified as lower uterine segment cancers.^17^, ^19^, ^20^, ^21^, ^22^, ^23^ In our study, we identified one carrier of a pathogenic variant in a Lynch Syndrome gene as having HPV‐negative adenocarcinoma of the cervix and a variant in *MSH6*. The other two carriers of pathogenic missense variants in Lynch Syndrome genes, *MLH1* and *MSH2*, in our study had HPV16‐positive adenocarcinoma or squamous cervical cancer, respectively. The data indicate that classical Lynch Syndrome gene variants are uncommon in hospital‐based series of cervical cancer patients. This is consistent with Wen et al. who identified only one carrier of a *MSH2* gene variant among 358 Chinese cervical cancer patients.^14^ Another seven carriers in our study were identified with pathogenic variants in *MUTYH*, including five with missense variant p.G368D. The association of MMR gene variants with adenocarcinoma was partially driven by this variant. *MUTYH* encodes a base excision repair protein that interacts with the MMR system, and *MUTYH* variants have been associated with an increased susceptibility of colorectal cancer in a recessive manner. However, all carriers in our sequencing study were heterozygous only, and larger studies will be needed to clarify whether *MUTYH* gene variants contribute to cervical cancer susceptibility.

Apart from the potential role of HDR and MMR gene variants as risk modulators for cervical cancer, their prevalence could be of therapeutic relevance. In particular, the presence of HDR gene variants may offer the opportunity of targeted therapy with PARP inhibitors such as olaparib, an option that has been successful for breast and ovarian cancers with HDR deficiency. It will also be interesting to assess whether HDR gene mutated cervical cancers respond differently to platinum‐based therapy. On the other hand, MMR deficiency and MSI may offer further treatment options with Programmed cell death protein (PD‐1)/ Pro PD‐1/programmed death‐ligand 1 (PD‐L1) inhibitors because such tumours may have an increased mutational burden and excess production of tumour neoantigens.^24^ Some studies showed longer progression‐free survival with pembrolizumab or cemiplimab in persistent, recurrent or metastatic cervical cancer.^25^ Furthermore, an ongoing clinical trial (NCT05838768) aims to exploit an inhibitor of Werner helicase, HRO761, against microsatellite‐unstable cancers.^26^ Since this helicase also controls proliferation and DNA replication of HPV‐infected cells, its therapeutic use in cervical cancer remains to be clarified. According to our study, pathogenic germline variants in MMR genes are not common in cervical cancer, though this does not preclude their somatic occurrence due to other mechanisms of genomic instability.

In conclusion, our study has assessed the prevalence and potential role of pathogenic HDR and MMR gene variants in a large consecutive series of cervical cancer patients. While MMR gene variants were rare, our results indicate that a small but relevant proportion of cervical cancer patients are carriers of germline HDR gene variants, including several in known breast and ovarian cancer risk genes. Larger studies will be needed to exclude that such variants occur just by coincidence. These variants do not explain HPV‐negative cancer, but may contribute to the risk of invasive cancer, and they may be exploitable in future therapeutic managements.

## AUTHOR CONTRIBUTIONS

**Lara Kokemüller:** Formal analysis; investigation; methodology; validation; visualization; writing – original draft; writing – review and editing. **Dhanya Ramachandran:** Data curation; formal analysis; investigation; methodology; writing – review and editing. **Peter Schürmann:** Investigation; methodology; writing – review and editing. **Robert Geffers:** Formal analysis; investigation; methodology; writing – review and editing. **Matthias Jentschke:** Data curation; resources; writing – review and editing. **Gerd Böhmer:** Data curation; resources; writing – review and editing. **Hans‐Georg Strauß:** Data curation; resources; writing – review and editing. **Christine Hirchenhain:** Data curation; resources; writing – review and editing. **Monika Schmidmayr:** Data curation; resources; writing – review and editing. **Florian Müller:** Data curation; resources; writing – review and editing. **Peter A. Fasching:** Data curation; resources; writing – review and editing. **Alexander Luyten:** Data curation; resources; writing – review and editing. **Norman Häfner:** Data curation; resources; writing – review and editing. **Peter Hillemanns:** Data curation; funding acquisition; resources; supervision; writing – review and editing. **Thilo Dörk:** Conceptualization; data curation; formal analysis; funding acquisition; project administration; supervision; writing – original draft; writing – review and editing.

## FUNDING INFORMATION

Dhanya Ramachandran received intramural funding from the Tumorstiftung at the Comprehensive Cancer Center Lower Saxony. Thilo Dörk and Peter Hillemanns received funding from the Bruno and Helene Jöster Foundation (Grant no. 2021.142.1).

## CONFLICT OF INTEREST STATEMENT

Peter Fasching reports personal fees from Novartis; grants from Biontech, grants and personal fees from Pfizer, personal fees from Daiichi‐Sankyo, Astra Zeneca, Eisai, Merck Sharp & Dohme; grants from Cepheid, personal fees from Lilly, SeaGen, Roche, Agendia, Gilead, Mylan, Menarini, Veracyte and GuardantHealth, during the conduct of the study and from Translational Research in Oncology (TRIO). These funders had no influence on the design or results of the study. The other authors declare that the research was conducted in the absence of any commercial or financial relationships that could be construed as a potential conflict of interest.

## ETHICS STATEMENT

The patients gave written consent and the study was approved by the Ethics commission at Hannover Medical School (votes no. 441 and 10737).

## Supporting information


**Data S1:** Supporting information.

**Table S2:** List of primers used for multiplex amplification.

**Table S3:** Coverage information per sample.

## Data Availability

Genomic coordinates and primer sequences are available as Supporting Information. Other data that support the findings of this study are available from the corresponding author upon request.

## References

[1] Arbyn M , Weiderpass E , Bruni L , et al. Estimates of incidence and mortality of cervical cancer in 2018: a worldwide analysis [published correction appears in *Lancet Glob Health*. 2022;10(1):e41]. Lancet Glob Health. 2020;8(2):e191‐e203. doi:10.1016/S2214-109X(19)30482-6 31812369 PMC7025157

[2] zur Hausen H . Papillomaviruses and cancer: from basic studies to clinical application. Nat Rev Cancer. 2002;2:342‐350. doi:10.1038/nrc798 12044010

[3] Scarth JA , Patterson MR , Morgan EL , Macdonald A . The human papillomavirus oncoproteins: a review of the host pathways targeted on the road to transformation. J Gen Virol. 2021;102(3):001540. doi:10.1099/jgv.0.001540 33427604 PMC8148304

[4] Stanley M . Immunobiology of HPV and HPV vaccines. Gynecol Oncol. 2008;109(2 Suppl):S15‐S21. doi:10.1016/j.ygyno.2008.02.003 18474288

[5] Yoshida H , Shiraishi K , Kato T . Molecular pathology of human papilloma virus‐negative cervical cancers. Cancers (Basel). 2021;13(24):6351. doi:10.3390/cancers13246351 34944973 PMC8699825

[6] Lee JE , Chung Y , Rhee S , Kim TH . Untold story of human cervical cancers: HPV‐negative cervical cancer. BMB Rep. 2022;55(9):429‐438. doi:10.5483/BMBRep.2022.55.9.042 35725012 PMC9537028

[7] Hemminki K , Dong C , Vaittinen P . Familial risks in cervical cancer: is there a hereditary component? Int J Cancer. 1999;82(6):775‐781. doi:10.1002/(sici)1097-0215(19990909)82:6<775::aid-ijc1>3.0.co;2-v 10446440

[8] Yamamoto H , Hirasawa A . Homologous recombination deficiencies and hereditary tumors. Int J Mol Sci. 2021;23(1):348. doi:10.3390/ijms23010348 35008774 PMC8745585

[9] Olave MC , Graham RP . Mismatch repair deficiency: the what, how and why it is important. Genes Chromosomes Cancer. 2022;61(6):314‐321. doi:10.1002/gcc.23015 34837268

[10] Lynch HT , Lanspa S , Shaw T , et al. Phenotypic and genotypic heterogeneity of Lynch syndrome: a complex diagnostic challenge. Fam Cancer. 2018;17(3):403‐414. doi:10.1007/s10689-017-0053-3 29071502

[11] Ramachandran D , Schürmann P , Mao Q , et al. Association of genomic variants at the human leukocyte antigen locus with cervical cancer risk, HPV status and gene expression levels. Int J Cancer. 2020;147(9):2458‐2468. doi:10.1002/ijc.33171 32580243

[12] Karczewski KJ , Solomonson M , Chao KR , et al. Systematic single‐variant and gene‐based association testing of thousands of phenotypes in 394,841 UK biobank exomes. Cell Genom. 2022;2(9):100168. doi:10.1016/j.xgen.2022.100168 36778668 PMC9903662

[13] Sun KY , Bai X , Chen S , et al. A deep catalogue of protein‐coding variation in 983,578 individuals. Nature. 2024;631(8021):583‐592. doi:10.1038/s41586-024-07556-0 38768635 PMC11254753

[14] Wen H , Xu Q , Sheng X , Li H , Wang X , Wu X . Prevalence and landscape of pathogenic or likely pathogenic germline variants and their association with somatic phenotype in unselected Chinese patients with gynecologic cancers. JAMA Netw Open. 2023;6(7):e2326437. doi:10.1001/jamanetworkopen.2023.26437 37523182 PMC10391307

[15] Thompson D , Easton DF . Breast cancer linkage consortium. Cancer incidence in BRCA1 mutation carriers. J Natl Cancer Inst. 2002;94(18):1358‐1365. doi:10.1093/jnci/94.18.1358 12237281

[16] Kutler DI , Wreesmann VB , Goberdhan A , et al. Human papillomavirus DNA and p53 polymorphisms in squamous cell carcinomas from Fanconi anemia patients. J Natl Cancer Inst. 2003;95(22):1718‐1721. doi:10.1093/jnci/djg091 14625263

[17] Antill YC , Dowty JG , Win AK , et al. Lynch syndrome and cervical cancer. Int J Cancer. 2015;137(11):2757‐2761. doi:10.1002/ijc.29641 26077226 PMC4573262

[18] Carnevali I , Di Lauro E , Pensotti V , et al. HPV nonrelated endocervical adenocarcinoma in hereditary cancer syndromes. Tumori. 2020;106(6):NP67‐NP72. doi:10.1177/0300891620936752 32635821

[19] Jenkins D , Molijn A , Kazem S , et al. Molecular and pathological basis of HPV‐negative cervical adenocarcinoma seen in a global study. Int J Cancer. 2020;147(9):2526‐2536. doi:10.1002/ijc.33124 32474915

[20] Stolnicu S , Barsan I , Hoang L , et al. International Endocervical Adenocarcinoma Criteria and Classification (IECC): a new pathogenetic classification for invasive adenocarcinomas of the Endocervix. Am J Surg Pathol. 2018;42(2):214‐226. doi:10.1097/PAS.0000000000000986 29135516 PMC5762258

[21] Young RH , Clement PB . Endocervical adenocarcinoma and its variants: their morphology and differential diagnosis. Histopathology. 2002;41(3):185‐207. doi:10.1046/j.1365-2559.2002.01462.x 12207781

[22] Moat M , O'Donnell RL , McCluggage WG , Ralte A , Edmondson RJ . Gastric‐type adenocarcinoma of the cervix in a patient with Lynch syndrome: a case report. Gynecol Oncol Rep. 2014;10:41‐43. doi:10.1016/j.gynor.2014.03.002 26082936 PMC4458744

[23] Seay K , Bustamante B , Khutti S , Frimer M . A case of non‐HPV related primary endometrioid adenocarcinoma of the cervix. Gynecol Oncol Rep. 2020;32:100579. doi:10.1016/j.gore.2020.100579 32405523 PMC7212174

[24] Le DT , Durham JN , Smith KN , et al. Mismatch repair deficiency predicts response of solid tumors to PD‐1 blockade. Science. 2017;357(6349):409‐413. doi:10.1126/science.aan6733 28596308 PMC5576142

[25] Colombo N , Dubot C , Lorusso D , et al. Pembrolizumab for persistent, recurrent, or metastatic cervical cancer. N Engl J Med. 2021;385(20):1856‐1867. doi:10.1056/NEJMoa2112435 34534429

[26] Ferretti S , Hamon J , de Kanter R , et al. Discovery of WRN inhibitor HRO761 with synthetic lethality in MSI cancers. Nature. 2024;629(8011):443‐449. doi:10.1038/s41586-024-07350-y 38658754 PMC11078746

