# Supplementary material for: Germline variants of homology‐directed repair or mismatch repair genes in cervical cancer
Source: Int J Cancer. 2024 Oct 23;156(4):700–10. doi: 10.1002/ijc.35221 (PMC11661519; doi:10.1002/ijc.35221)
Supplement: Supplementary file 1 — Data S1: Supporting information. [file IJC-156-700-s001.pdf]

Supplementary Information

Germline variants of homology-directed repair or mismatch repair genes in cervical cancer

Lara Kokemüller, Dhanya Ramachandran, Peter Schürmann, Robert Geffers, Matthias Jentschke, Gerd Böhmer, Hans Georg Strauss, Christine Hirchenhain, Monika Schmidmayr, Florian Müller, Peter Fasching, Alexander Luyten, Norman Häfner, Peter Hillemanns, Thilo Dörk

List of content:

- **Supplementary Figure S1:** Sanger sequencing results documenting six novel variants in *BRCA2*, *SLX4, FANCM*, *MLH1* and *MSH2*, respectively. Left panel: Wildtype sequence; right panel: heterozygous mutant. Chromosomal position refers to the GRCh37.p13 genome build.
- **Supplementary Table S1:** Clinical characteristics of 728 patients with cervical cancer or dysplasia that were included into the targeted sequencing study. LSIL, low-grade squamous intraepithelial lesion; HSIL, high-grade squamous intraepithelial lesion. Mean age at diagnosis is provided with 95% confidence interval. HPV status is listed as positive (HPV+), negative (undetected) or untested (unknown).
- **Supplementary Table S2** (uploaded as separate xls.file)**:** List of primers used for multiplex amplification of the coding region in the named genes using Fluidigm Access Arrays. Primer assays are listed with assay ID and assay name provided by Fluidigm, sequences of forward and reverse primers, amplicon size in base-pairs, and the chromosomal region covered (GRCh38.p14).

- **Supplementary Table S3** (uploaded as separate xls.file): Total number of reads and coverage per sample. Number of reads are provided as sequenced reads and uniquely mapped reads, respectively. Coverage is provided as median and mean coverage as well as first and third quartile coverage, and Percentage of targeted bases with coverage >=15 or with coverage >=200, respectively.

- **Supplementary Table S4:** List of primers used for PCR amplification and validation Sanger sequencing of identified pathogenic variants. Primer sequences listed in 5´-3´direction, amplicon size given in base-pairs.

- **Supplementary Table S5**: Total number of carriers of truncating variants in 12 HDR genes sequenced in the Cervigen cases with cervical cancer and compared to population-based controls from the UK Biobank (UKB) as accessed through the GeneBass portal and to population-based control from the Regeneron Genetic Center Million Exomes (RGC-ME) dataset as accessed through the RGC Million Exome Variant browser. Statistical analyses were performed only for the cumulative frequencies due to low numbers of single-gene variant carriers in the Cervigen series. P-value is provided from a Fisher´s exact test (2 df). CI, confidence interval; df, degrees of freedom.

Supplementary Figure S1: Identification of six novel pathogenic variants in HDR and MMR genes

<u>HDR genes:</u>

Wildtype control **Heterozygous variant carrier**

BRCA2 [chr13:32914769] c.6277delCA

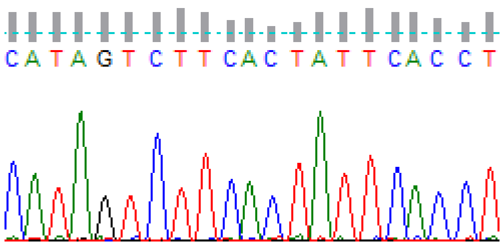

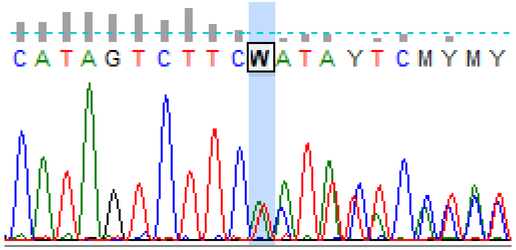

SLX4 [chr16:3639616] c.4024delA

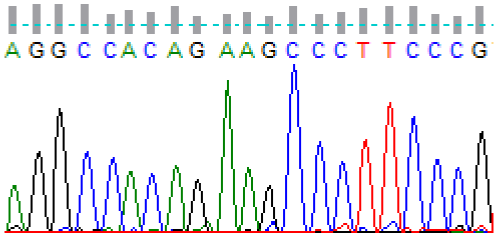

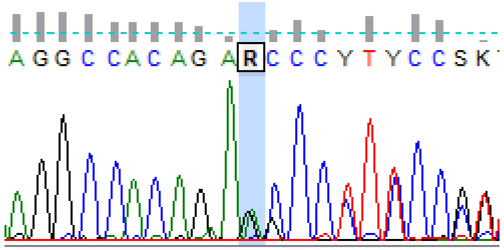

SLX4 [chr16:3641087] p.Met851Arg

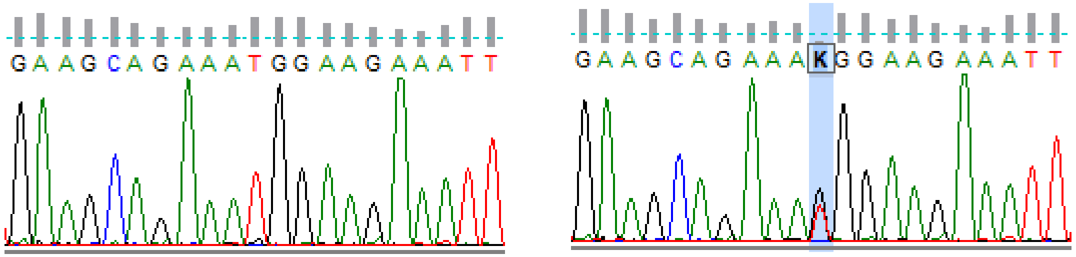

FANCM [chr14:45606344] p.Met194Thr

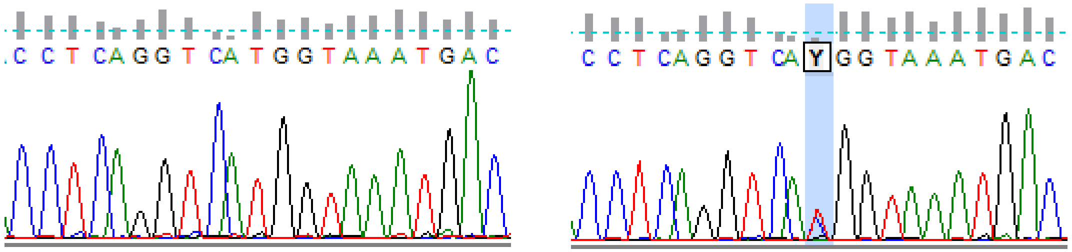

MMR genes:

Wildtype control **Heterozygous variant carrier**

MLH1 [chr3:37083793] T>G p.Phe568Val

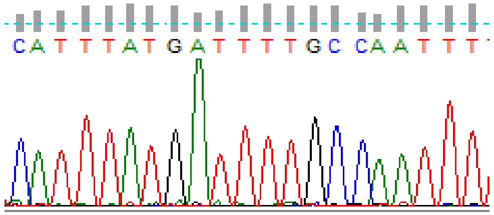

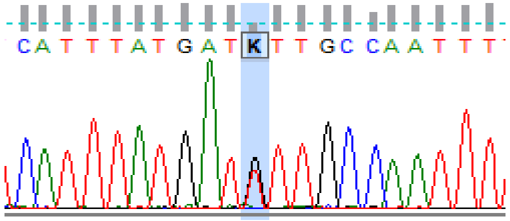

MSH2 [chr2:47637405] A>G p.Asp180Gly

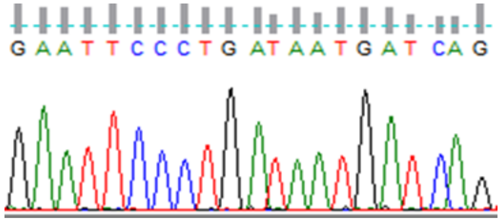

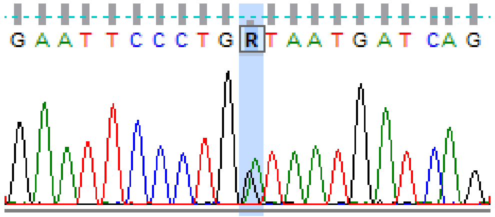

Supplementary Table S1: Study characteristics

**Stratum**	**Number of patients**	**Mean age at diagnosis**	**HPV+/ HPV undetected/ HPV unknown**
**total**	**728**	**42.8 (41.7 – 43.8)**	**554 / 58/ 116**
- **dysplasias**	**224**	**32.9 (31.6 – 34.2)**	**182/ 40/ 2**
-- LSIL	15	28.7 (24.4 – 33.1)	10/ 5 / 0
-- HSIL	209	33.2 (31.9 – 34.6)	172 / 35 / 2
- **invasive**	**504**	**47.1 (45.9 – 48.3)**	**372 / 18 / 114**
-- squamous	325	48.6 (47.1 – 50.1)	287 / 12 / 26
-- adenocarcinoma	167	44.6 (42.7 – 46.5)	73 / 6 / 88
-- other/ mixed	12	41.9 (31.5 – 52.2)	12 / 0 / 0

Supplementary Table S4: List of primers used for PCR amplification and Sanger sequencing

Gene	Forward Seq Primer (5´-3´)	Reverse Seq Primer (5´-3´)	Amplicon size (bp)
BARD1	CTC ACC TGT ACT GTC AAA CTC	GTT CTG GGT GTA GAT TCA ATG	288
BARD1	AGT CTG CTT TAT CAC ACA CC	TTG CAA TCA TGG GCA CCT G	240
BARD1	ACC TGA CAG CTC ATT GTC ATG	GTT AGA ATA ACC TCT GCT CC	257
BRCA1	GGA AAG TAT CGC TGT CAT GTC	TCT ACC AGG CAT ATT CAT GCG	247
BRCA1	TAG CTT CTT AGG ACA GCA CTT C	TGC AAT TCT GAG GTG TTA AAG G	200
BRCA1	CAA TCC TAG CCT TCC AAG AG	AAT GCT GCA CAC TGA CTC AC	258
BRCA1	AAC GGA GCA GAA TGG TCA AG	CTG ACT ACT AGT TCA AGC GC	270
BRCA2	CTG CTA TAC GTA CTC CAG AAC	GAG TTT ACA CAG TGC TCT GG	268
BRCA2	TAT TAG TGT CGC CAA AGA GTC	CCG CTA GCT GTA TGA AAA CC	282
BRCA2	CTC CAC CCT ATA ATT CT G AAC	ACT GAT TTG CCC AGC ATG AC	310
BRCA2	GAT TTA TCC TGT TTA GAC CCT G	TCT GGA GTG CTT TTT GAA GCC	338
BRCA2	GAA G GC ATT TCA GCC ACC AA	TCA CAT TCT TCC GTA CT G GC	330
BRCA2	CTG TTT GCT CAC AGA AGG AG	GAC AGA GGT ACC TGA ATC AGC	290
BRCA2	TTG AAA TGA CTA CTG GCA C	CTG GCC AGA TAA TTT AAG AC	221
BRCA2	CTC ATC TGC AAA TAC TTG TGG	GTT CTG GAG TAC GTA TAG CAG	206
BRIP1	CTG GAA GAT GCA GAG TTT G	CTT AAG GTT TTG ATG GCC TAC	380
BRIP1	CAT GAC CCA ACT AAT CTC CAC	CCT GAA AAA ACC ACT CTG GC	226
BRIP1	AGA GGC ACT ATT CTC TGA TG	TGT TAG CTA GGA GCA GAA AG	237
BRIP1	AGA AGC CTA GTT AAC CAA AG	ATA ACA TCG AGG ACT GTG C	260
BRIP1	CTG GAA GAT GCA GAG TTT G	CTT AAG GTT TTG ATG GCC TAC	380
BRIP1	ACT TCA GAT GGA GAG AGA CC	TAA GTT AGC GAC AGC ATG GC	283
ERCC4	TGG AGT ACG AGC GAC AGC TG	GTC ATC GCG TAG TGT CAG GG	290
ERCC4	GCC CTG TAT TAA ATA GCC TAC	GTC AAC CAC AAG TAT CCT AC	188
ERCC4	GAG GTG CCT TGT TTC AGG AG	ATC AGG CTG TGG CTT GCT TTG	158
ERCC4	CCC GAG GTG CCT TGT TTC AG	TAA CGT GGT GCA TCA AGG AG	300
ERCC4	CAT ATG TAC TGA TGC TCG TG	GAT CTC AGT GTT CAT TTG CC	398
ERCC4	ACA AAG AAC GGG CTT CTA CC	TTC AAG CTG CCG AAC AAA GG	356
ERCC4	TCA GGA GAT CTC CAG CAA TG	CAG AAT CTG CTG TAA TGG CC	179
ERCC6	GTG TCT GAA CAT CTG ATC CAG	CAA AGG CGG TTT TTC AAA TCC	206
ERCC6	AGA CTG CCT GGG AAG AGC TC	TCA TGT CTG ACT CCC AAG GTC	381
FANCM	AAC TCG ACG TGC AGT AAT GC	TGG TGT GGT AGA CTG GAC TTC	358
FANCM	AAG TAA CAG GCA GGT CCT TC	GCC TTT CCA AAG AGA ATG CC	304
FANCM	CAG GGT CTA CAC AAG CTT CC	GGC AAT AAG CAT AGT TTC CG	174
FANCM	TCT GAT GAG CCA AGT CTC TG	TGA CAC AGG TAA AGA TTC GC	264
FANCM	AAC TCG ACG TGC AT AAT GC	GGT GTG GTA GAC TGG ACT TC	357
FANCM	GGA CGA TGA TGT GTT GCT TG	GAT CTG CTG TGT CAC CAA G	298
FANCM	GTT GTG TCT AGA GAA TGG CG	CAA GCC TCG ATC TGC TGT G	261
FANCM	CCT GGA GAA AAG GAA ACC ATT	TGA AGT ACT ACG TGT ACG TGC	282
MLH1	CCA TTC TGA TAG TGG ATT C	TGA GCA GCT TGG ATT ACA G	479
MLH1	GTG AG ATA AAA CCC TAG CC	GCT GAG CAC AGA CTT AGG AC	269
MLH1	CTG TTA ACC AGA TTC CAC AGC	CAA TAC AGC AAC TAT CCT TAG AA	334
MSH2	GAA GTC CAG CTA ATA CAG TGC	CCA ATC ATT CTC CTT GGA TG	210
MSH2	GTC AGC TTC CAT TGG TGT TG	CAA TTT GCT TAC CTG TCT CAG	235
MSH6	ATG TGT AGC TCA TGA TAG C	TCT GTG CCA CAA TGG TGA G	325
MUTYH	CAT GGT AGG TCC CGT TTC TC	GCC AGT AGT ACC ACC CTG AG	352
MUTYH	TGG TCA ACT TCC CCA GAA AGG	TCT TGT TAC TCA TGC CAC TGC	168
MUTYH	ATT CCG CTG CTC ACT TAC CT	CAG AGG CCC AAC TCA GGT AC	275
MUTYH	CTC TGC ACC AGC AGA ATT TGG	AGC CCT CTT GGC TTG AGT AG	221
MUTYH	CCT GAT TGG AGT GCA AGA CTC	GGC TAT AGA AGT GGC CTA CAC	281
MUTYH	ACA GCT CTT AGC CTC AGG GAG	TGT TAC TCA TGC CAC TGC CC	456

MUTYH	CAG GA G AT G TAC T GA CCA GC	T G G TAT T GC A G G CCT CTG TC	269
MUTYH	TCT GCT TCA CAG CAG TGT TCC	CCT GAG GCT AAG AGC TGT TC	284
PALB2	CGA GTT TGG CCT TTT GGG ATG	GGC TCC ATT TCA TAG GGA TGG	301
PALB2	TGC CAG ACA TCC TAA TTT CAC	GGA AAG TGA GAT TCT AAG TC	293
PALB2	AGT GAC ACT CTT GAT GGC AG	TCC TCT GGC AAT TGG ACA TG	190
PMS2	CCA GTC CTG AAC TCC TAG CC	ACC TGA AGT GCT AGA AGA CAG	476
PMS2	TGC TGT GCT T GAT GAT GTA AC	TCG CA G GAA CAT GTG GAC TC	274
PMS2	GCC ATC ACT ACC T GC TTC T G	GAT GTG AGA ACC TTG CGT TG	275
PMS2	ATC AGG AGC TGG GCT GAG AC	AAC GTG TTT GTC AAG TCA TGG	294
POLD1	AGC ACT GCT CCC AGC CAA TG	ACA CAT GCT GAA TTG GGC AC	261
POLD1	A GC T G G GAA ATA CGC CCT GAG	GAC TTG GCA CAT CAA TGA GG	289
POLE	CAG GTA TGC TGT GTT CAA TG	CTT GGC TAC AGA GCC ATA C	163
POLE	CAT GAG ATG TGG TGA CAG C	GCA TTA GAG CCT GAC CTG C	262
RAD51	ACT ACT CGG GTC GAG GTG AG	TGA TGA TCT CAG GCA CTG ATG	181
RAD51B	TTT A GA TTA CA G GTC CAC C	ACC TTT CAG CAC TAA ATG C	144
RAD51B	GTG CTT TAT GTG CAG ATT C	CTA CCT CAA TCT TCT CTG TC	294
RAD51B	TTA GAT TAC AGG TCC ACC AGG	ATG AGC CCA GAT CGT GCC AC	314
RAD51C	CCT AAC TTG TCA TTA TCT GG	TGC AGG CAG TAG CAA GGT C	209
RAD51C	TAC AAG ACT GCG CAA AGC TG	TGC CTC AGC TTG CCA TCA G	323
RAD51C	TAG CAG GTG AGC CTG CGA TG	TGC CTC AGC TTG CCA TCA G	191
RAD51D	AGT TCC AGA CCT GCC ATT AGG	ATT TGG CCA GAC ACG CCA TG	301
RAD51D	AGT TCC AGA CCT GCC ATT AGG	ATT TGG CCA GAC ACG CCA TG	301
SLX4	CGT GGT CCT TGG ATT TCA AC	GCG ATG ACT GTC GAT GTT TC	220
SLX4	TCA GTA GGA AAC AGG GAA GG	GTC CCC TGA GAT GGG ATG T	213
SLX4	AGC AGA AGC CGT GAC TGT TC	TGC TCA TCG TCA CTG TCT CC	416
SLX4	TGA GCT CGT TCA CCT GTG C	AGC GCA CTG TCC CAT CTT C	442
SLX4	CAT CCT CAC GCT GTC TAA AG	TCA TCC GAG TCC AGT AAG AG	216
SLX4	GTC TCC AGC TGT GCC ATC AA	GAG TCC AGT AAG AGG ATG AC	249
SLX4	GCT CTT CTC ATC AAC TCA GG	ACG ACC CAC TTG TGT GAT G	209
SLX4	CTG ATC AGT AAA CCC TCT GTG	GAG CTG TTA ACC TGC CAG TC	264
SLX4	ATT GGT CCT ACA GCG AAT GC	GAG ACT CTT CAT TCT CTG GC	308

Supplementary Table S5: Truncating HDR gene variants in cervical cancer cases and population-based controls

Gene	Cervical Cancer (n=1,456)	Controls UKB (n=426,760)	Controls RGC-ME (n=1,643,940)
BARD1	1	95	634
BRCA1	2	229	1,237
BRCA2	2	673	2,454
BRIP1	3	380	1,548
ERCC4	0	92	501
FANCM	4	1,032	3,339
PALB2	0	394	1,399
RAD51	0	5	12
RAD51B	0	91	43*
RAD51C	0	92	420
RAD51D	1	146	417
SLX4	2	305	232
**Total**	**15**	**3,534**	**12,193**
**Odds ratio (Cervical cancer vs controls)**	**.**	**1.25 (0.70-2.07)**	**1.39 (0.78-2.31)**
**P (2df, exact)**	**.**	**p=0.382**	**p=0.217**

Legend to Supplementary Table S5: Comparison of allele frequencies for truncating variants in 12 HDR genes in the cervical cancer series and two large public datasets, the UK Biobank ("Controls UKB") and the Regeneron Genetics Center Million Exome ("Controls RGC-ME") databases. Allelic counts are listed by gene and summarized as total. For *BRCA2*, truncating variants in the last exon were not considered pathogenic. For RGC-ME, the total number n indicates the median of all sequenced control chromosomes. Odds ratios and p-values were determined using Fisher´s exact test. * Variants in *RAD51B* were flagged as low-quality in the RGC-ME database.
